# ASC contributes to metastasis of oral cavity squamous cell carcinoma

**DOI:** 10.18632/oncotarget.10317

**Published:** 2016-06-29

**Authors:** Chi-Sheng Wu, Kai-Ping Chang, Chun-Nan OuYang, Huang-Kai Kao, Chuen Hsueh, Lih-Chyang Chen, Hsiao-Yun Cheng, Ying Liang, Willisa Liou, Chih-lung Liang, Yu-Sun Chang

**Affiliations:** ^1^ Chang Gung Molecular Medicine Research Center, Chang Gung University, Taoyuan, Taiwan; ^2^ Graduate Institute of Basic Medical Sciences, Chang Gung University, Taoyuan, Taiwan; ^3^ Department of Otolaryngology-Head & Neck Surgery, Chang Gung Memorial Hospital, Taoyuan, Taiwan; ^4^ Department of Plastic and Reconstructive Surgery, Chang Gung Memorial Hospital, Taoyuan, Taiwan; ^5^ Department of Pathology, Chang Gung Memorial Hospital, Taoyuan, Taiwan; ^6^ Department of Medicine, Mackay Medical College, New Taipei City, Taiwan; ^7^ Department of Anatomy, Chang Gung University, Taoyuan, Taiwan; ^8^ School of Medicine, Chung Shan Medical University, Taichung, Taiwan

**Keywords:** ASC, OSCC, metastasis

## Abstract

ASC (Apoptosis-associated Speck-like protein containing a CARD) acts as a platform protein in the inflammasome cascade of some cancer types. However, its potential involvement in OSCC (oral cavity squamous cell carcinoma) has not yet been determined. Here, we investigated the potential role of ASC in OSCC. RT-qPCR analysis of 20 paired tumor and adjacent normal tissue samples revealed that the mRNA levels of ASC, along with IL-1β, CASP1, and NLRP3 in ASC-associated NLRP3 inflammasome were significantly elevated in OSCC tissues. Immunohistochemical staining of these four proteins in 111 clinical specimens revealed that high-level expression of ASC was significantly associated with tumor stage, node stage (*p*=0.001), overall stage (*p*<0.001), extracapsular spread (*p*<0.001), perineural invasion (*p*=0.004) and tumor depth (*p*<0.001). Kaplan-Meier survival analysis further revealed that high-level ASC expression was correlated with poorer overall survival (*p*=0.001), disease-specific survival (*p*<0.001) and disease-free survival (*p*<0.001). Studies using OSCC cell lines indicated that high-level ASC expression enhanced cell migration and invasion, and experiments using an orthotropic nude mouse model confirmed that ASC overexpression induced metastasis of OSCC cells. This is the first report to show that ASC contributes to OSCC metastasis, and that high-level ASC expression is a marker for poor prognosis in OSCC patients.

## INTRODUCTION

Oral cavity cancer is the most common head-and-neck cancer, which accounts for approximately 3% of all newly diagnosed cancer cases [1, 2]. Most oral cavity cancers are oral cavity squamous cell carcinomas (OSCCs), which are locally aggressive and show moderate locoregional recurrence and survival rates [3–5]. Despite modern improvements in the treatment modalities, which include surgery, radiotherapy and chemotherapy, the 5-year overall survival rate of OSCC patients is only ~ 60% [6–8]. OSCC can arise from various locations in the oral cavity, including the tongue, buccal area, gingiva, lip, floor of mouth, and hard palate. The disease develops from precancerous dysplastic lesions through multistep processes of carcinogenesis that usually involves chronic exposure to carcinogens (e.g., tobacco exposure, alcohol intake, and inflammation) [9–12]. In Taiwan, alcohol, betel nut chewing and smoking are considered to be the major risk factors for OSCC. These factors have been shown to induce NLRP3 inflammasome activity and/or IL-1β activation in other cell types and diseases [13–15], but the role of the NLRP3 inflammasome in OSCC has not yet been explored.

Apoptosis-associated Speck-like protein containing a CARD domain [ASC; also called PYD and CARD domain-containing protein (PYCARD), target of methylation-induced silencing (TMS1) [16], is the platform protein of inflammasomes. It plays a key role in activating the inflammasome [17] by interacting via its PYD domain with pattern recognition receptors (e.g., NLRP3), and via its CARD domain with pro-caspase-1, leading to caspase-1 activation; this inflammasome activation promotes maturation of the cytokines, IL-1β and IL-18 [18]. The maturation of IL-1β by an activated inflammasome is an important mechanism for tumor pathogenesis in melanoma [19], colorectal cancer [20, 21], and nasopharyngeal carcinoma (NPC) [22]. Indeed, accumulating information suggests that ASC may play multiple roles in cancer. For example: it can function as a tumor suppressor; its expression is decreased by promote methylation in cancers such as melanoma [23], breast cancer [24], ovarian cancer [25], prostate cancer [26], colorectal cancer [27] and hepatocellular carcinoma [28]; it is reportedly involved in the progression of melanoma [29]; and our group showed that it is highly elevated in NPC tumor cells, where its expression correlates with local recurrence and the survival of patients [22]. These findings prompted us to explore the potential role of inflammasome proteins in OSCC, which is believed to be associated with local inflammation.

Here, we demonstrate for the first time that NLRP3 and its inflammasome-associated proteins, ASC, IL-1β, and CASP1, are highly expressed in OSCC tumor tissues, as examined by immunohistochemistry (IHC), Western blot and RT-qPCR analyses. We also demonstrate that high-level expression of ASC (but not the other three proteins) was significantly associated with various clinicopathological characteristics (*p*<0.001) and was correlated with poorer patients' survival (*p*<0.001). *In vitro* studies using OSCC cell lines and *in vivo* lymph node metastasis model of oral squamous cell carcinoma confirmed that ASC induced OSCC cell metastasis.

## RESULTS

### Overexpression of inflammasome genes in OSCC tumor tissues

**Figure 1:**
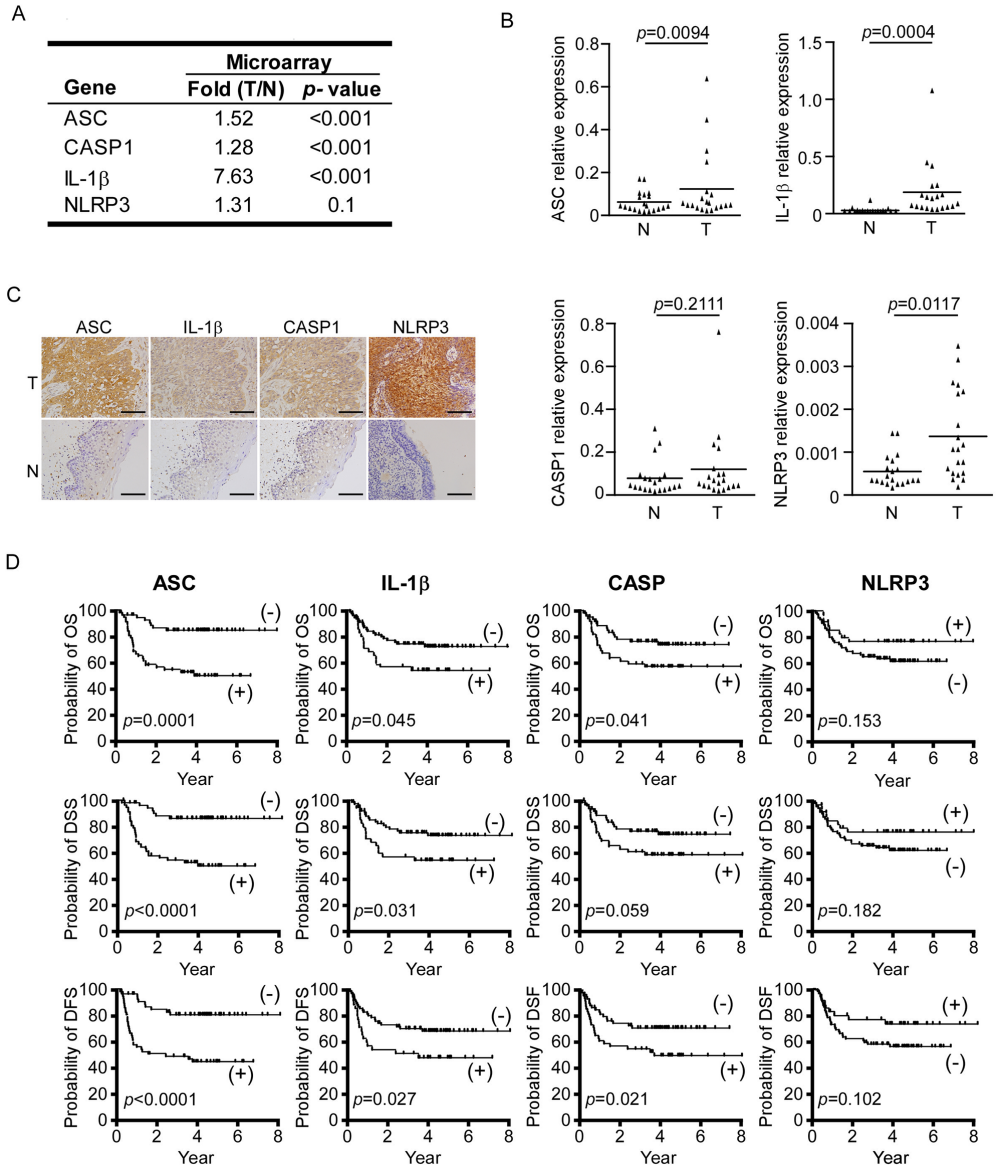
Inflammasome proteins are highly expressed in OSCC **A.** Microarray results for NLRP3 inflammasome-associated proteins. **B.** Relative mRNA expression levels of *asc*, *il-1b*, *casp1*, and *nlrp3* in tumor (T) and adjacent normal tissues (N). **C.** ASC, IL-1β, CASP1, and NLRP3 proteins are overexpressed in the tumor regions (T) compared to adjacent normal regions (N) of OSCC clinical samples. Magnification, X400; bar, 50 μm. **D.** Kaplan-Meier survival analysis of OSCC patients. The overexpressions of ASC, IL-1β, and CASP1 (but NLRP3) are associated with poor prognoses for OS (upper), DSS (middle) and DFS (lower). ASC positive (+), n=54; ASC negative (-), n=57; IL-1β positive (+), n=35; IL-1β negative (-), n=76; CASP1 positive (+), n=50; CASP1 negative (-), n=61.

We examined the expression of NLRP3 inflammasome-related components in 20 OSCC tumors and adjacent normal tissues using microarray analysis [30] (Supplementary Table S2 and Figure 1A), followed by RT-qPCR validation in an independent group of samples (n=20). RT-qPCR revealed that the expression levels of all four tested NLRP3 inflammasome-associated genes (*asc, casp1, il-1b*, and *nlrp3*) were highly elevated in the 20 OSCC tissues, by 1.89-fold, 1.7-fold, 46.31-fold and 3.76-fold, respectively (Figure 1B). We next used immunohistochemical (IHC) staining to evaluate the protein expression levels of ASC, IL-1β, CASP1 and NLRP3 in tumor cells using a second cohort of OSCC biopsy samples (n=111). Our results confirmed that these proteins were highly expressed in OSCC tumor cells, but were only weakly expressed in adjacent normal cells (Figure 1C). Clinical details on the enrolled patients are shown in Supplementary Table S1. Together, our results show that the NLRP3 inflammasome-related molecules are overexpressed in OSCC.

### Associations of ASC, IL-1β and CASP1 with clinicopathological manifestations

To investigate the potential clinical significance of the NLRP3 inflammasome in OSCC, we analyzed the associations of our IHC staining scores with the patients' clinicopathological characteristics. As shown in Table 1, high-level expression of ASC was significantly correlated with many clinicopathological characteristics, including tumor stage (*p*<0.001), node stage (*p*<0.001), overall stage (*p*<0.001), extracapsular spread (*p*<0.001), perineural invasion (*p*=0.004) and tumor depth (*p*<0.001). In addition, high-level expression of IL-1β was correlated with age (*p*=0.024) and overall stage (*p*=0.023); high-level expression of CASP1 was correlated with node stage (*p*=0.041), overall stage (*p*=0.006) and perineural invasion (*p*=0.013); and high-level expression of NLRP3 was correlated with extracapsular spread (*p*=0.020) and perineural invasion (*p*=0.013). These results indicate that ASC, IL-1β, CASP1, and NLRP3 are all highly associated with the clinicopathological characteristics of OSCC, and that ASC is significantly associated with all of the examined clinicopathological manifestations.

**Table 1:** Association of expression levels (immunohistochemical score) of three proteins increasing in the OSCC tumors with clinicopathological characteristics in 111 untreated OSCC patients

	No.	ASC		IL1β		CASP1		NLRP3	
		score*	*p*-value	score	*p*-value	score	*p*-value	score	*p*-value
**Gender**									
Female	14	107±55	0.234	111±58	0.809	112±47	0.311	160±40	0.264
Male	97	127±36		118±33		129±34		147±47	
**Age (yr)†**									
<49.5	55	120±35	0.139	111±35	0.024	127±34	0.760	151±46	0.760
>49.5	56	129±43		123±38		127±39		146±46	
**Tumor stage**									
1-2	65	114±40	0.001	115±41	0.274	123±38	0.075	151±52	0.323
3-4	46	138±34		123±30		133±33		145±37	
**Node stage**									
(-)	66	109±38	<0.001	112±37	0.132	121±36	0.041	149±46	0.798
(+)	45	146±30		125±36		136±35		148±46	
**Overall stage**									
I-II	42	100±36	<0.001	106±39	0.023	115±36	0.006	147±52	0.910
III-IV	69	139±34		124±34		134±34		149±42	
**Extracapsular spread**									
No	87	118±38	<0.001	114±36	0.139	124±35	0.158	153±45	0.020
Yes	24	148±37		129±37		137±37		130±46	
**Perineural invasion**									
No	77	117±40	0.004	113±35	0.212	121±36	0.013	154±50	0.013
Yes	34	142±31		128±40		140±34		135±34	
**Tumor depth^†^**									
<=8	58	110±38	<0.001	113±39	0.194	121±35	0.193	153±53	0.102
>8	53	140±34		122±34		133±37		144±37	

* Mean±SD;

† Median

### Associations of ASC, IL-1β and CASP1 overexpression with patient overall survival (OS), disease specific survival (DSS) and disease free survival (DFS)

We further evaluated whether the overexpression of ASC, IL-1β, CASP1, and/or NLRP3 was correlated with patients' OS, DSS, and DFS (Figure 1D). Survival analysis using Kaplan-Meier plots for high expression of ASC, IL-1β and CASP1 patients versus low expression of ASC, IL-1β and CASP1 patients, respectively. We revealed that the 5-year OS rates for the stratified patients with high expression of ASC, IL-1β and CASP1 were 84.5% versus 49.8% in ASC, 72.1% versus 54.1% in IL-1β, and 73.9% versus 57.1% in CASP1, respectively (*p*=0.0001, 0.045, and 0.041, respectively, by log-rank test); the 5-year DSS rates stratified patients with ASC, IL-1β and CASP1 were 86.0% versus 49.7% in ASC, 73.1% versus 54.1% in IL-1β, and 73.9% versus 58.2% in CASP1, respectively (*p*<0.0001, =0.031, and =0.059, respectively, by log-rank test); and the 5-year DFS rates stratified patients with ASC, IL-1β and CASP1 were 80.4% versus 44.8% in ASC, 68.4% versus 48.2% in IL-1β, and 71.3% versus 50.4% in CASP1, respectively (*p*<0.0001, =0.027, and =0.021, respectively). In contrast, NLRP3 overexpression was not found to correlate with survival (Figure 1D).

We next performed multivariate analysis using age, gender, overall stage, perineural invasion, and overexpression of the four target proteins as parameters in a Cox proportional regression model. Our results further indicated that upregulation of ASC was an independent predictor of OS, DSS, and DFS (*p* = 0.026, 0.013, and 0.042, respectively), whereas the other tested proteins were not found to predict these parameters (Table 2).

**Table 2:** Multivariate analysis on overall survival, disease-specific survival, and disease-free survival of patients with squamous cell carcinoma after treatment

Characteristics	Overall survival		Disease-specific survival		Disease-free survival	
	Hazards Ratio (95% Confidence Interval)	*p*-value	Hazards Ratio (95% Confidence Interval)	*p*-value	Hazards Ratio (95% Confidence Interval)	*p*-value
ASC overexpression						
Yes	1.000 (Reference)	0.026†	1.000 (Reference)	0.013†	1.000 (Reference)	0.042†
No	2.510 (1.116 − 5.646)		2.935 (1.251 − 6.886)		2.153 (1.026 − 4.519)	
IL-1β overexpression						
Yes	1.000 (Reference)	0.367	1.000 (Reference)	0.264	1.000 (Reference)	0.464
No	1.411 (0.668 −2.984)		1.547 (0.719 −3.330)		1.303 (0.641 −2.649)	
Caspase 1 overexpression						
Yes	1.000 (Reference)	0.110	1.000 (Reference)	0.141	1.000 (Reference)	0.097
No	1.806 (0.874 −3.732)		1.738 (0.833 −3.628)		1.795 (0.899 −3.585)	
NRLP3 overexpression						
No	1.000 (Reference)	0.380	1.000 (Reference)	0.446	1.000 (Reference)	0.242
Yes	0.684 (0.293 −1.599)		0.717 (0.305 −1.687)		0.618 (0.275 −1.386)	

* Median age is 49.5 y;

† statistically significant

### ASC contributes to the migration and invasion of OSCC cells

**Figure 2:**
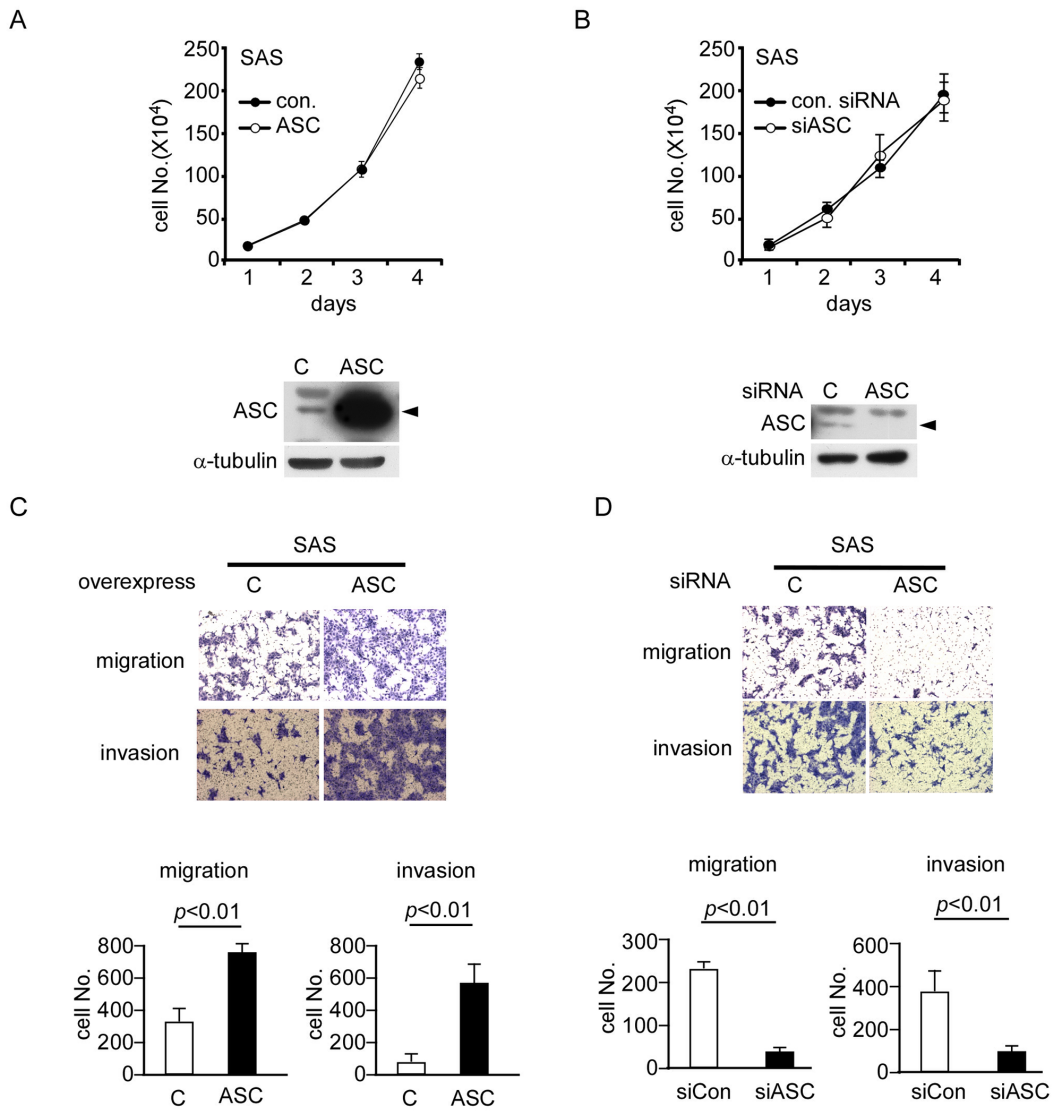
ASC promotes migration and invasion ability in OSCC cell lines **A** and **B.** Cell proliferation assays were performed on SAS cells overexpressing ASC or the control vector (A) or transiently transfected with control or ASC-targeting siRNAs (B) Western blot results show the expression levels of ASC in the SAS cells. **C.** Migration and invasion assays of ASC-overexpressing SAS cells show 2.3- and 6-fold increases in cell migration (top) and invasion (bottom), respectively. **D.** Migration and invasion assays of ASC-knockdown cells show ~75% decreases in migration (top) and invasion (bottom) compared with the vector control.

Since ASC was overexpressed in OSCC tumor cells, and this overexpression was highly associated with OSCC metastasis, we tested the effect of ASC protein overexpression/knockdown on the cell migration and invasion of OSCC cell lines *in vitro*. In SAS cells, ASC was overexpressed or knocked down by transfection with an ASC-expressing vector or an ASC-targeting siRNA, respectively, as confirmed by Western blot analysis (Figure 2A and Figure 2B). These alterations in the expression level of ASC did not affect the cell growth rate, as demonstrated in Figure 2A and 2B. However, the migration and invasion abilities were 2.3-and 6-fold higher in ASC-overexpressing cells compared to cells transfected with the vector control (Figure 2C). Moreover, ASC-knockdown SAS cells showed significant inhibitions of cell migration and invasion (both by ~75%) (Figure 2D). Similar results were also observed when we overexpressed ASC in low-expressing OECM1 cells (Supplementary Figure S1A) or knocked down ASC in high-expressing OC3 cells (Supplementary Figure S1B). Enhanced cell migration and invasion were observed in ASC-overexpressing OECM1 cells (Supplementary Figure S1C), whereas reductions of 30% and 60% were seen in the migration and invasion abilities, respectively, of ASC-knockdown OC3 cells (Supplementary Figure S1D). Together, these data indicate that ASC enhances the cell migration and invasion abilities of OSCC cell lines.

### ASC promotes lymph node metastasis in nude mice

**Figure 3:**
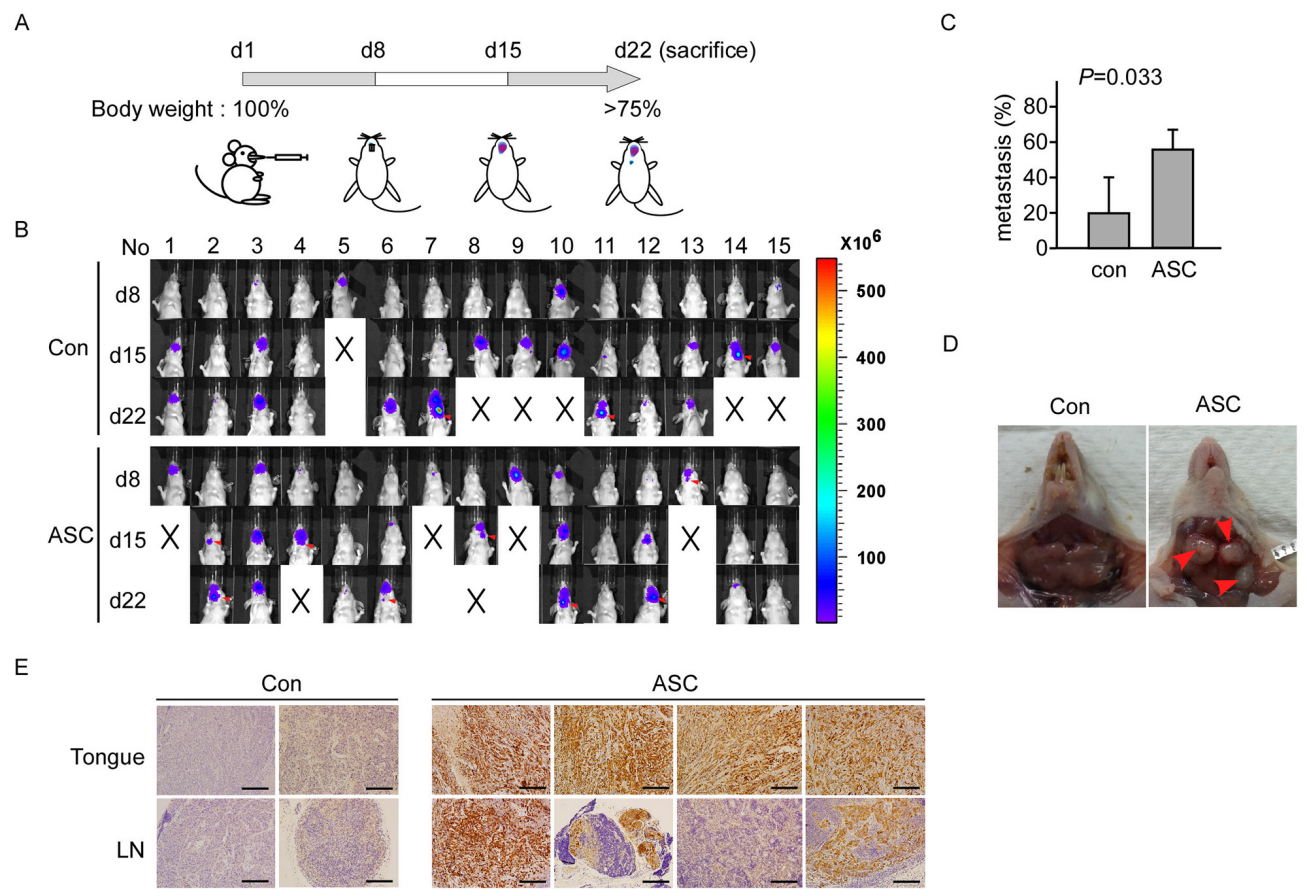
ASC promotes tumor metastasis in an animal model **A.** Design of the animal study. **B.** The IVIS visualization system used to track tumor growth and metastasis. Mice were inoculated with SAS_Luc2 or SAS_Luc2_ASC on day 1, and images were monitored and recorded on days 8, 15 and 22. Red arrows indicate the metastatic lymph node. **C.** Comparison of metastatic lymph node between SAS_Luc2 and SAS_Luc2_ASC mice, data shown as mean±SE from 3 independent experiments; 5 mice were tested in each group for each experiment (*p*=0.033). **D.** Image of lymph node metastasis in the cervical region of a SAS_Luc2_ ASC mouse. Red arrows indicate the metastatic lymph node. **E.** Histological image of tongue and lymph nodes sections in four representing SAS_Luc2_ASC-carrying mice, and in two SAS_Luc2 control mice as described in Results. ASC is highly expressed in SAS_Luc2_ASC mice, but not in SAS_Luc2 mice. Magnification, X200; bar, 100μm.

As our clinical observations have suggested that ASC could be associated with lymph node metastasis, we generated SAS cells that stably overexpressed ASC (SAS_Luc2_ASC) or vector control (SAS_Luc2), used them to inoculate nude mice, and examined ASC-associated metastasis in an oral cancer (tongue) model (Figure 3A; for details, see Materials and Methods). Nude mice were inoculated with SAS_Luc2 or SAS_Luc2_ASC cells (n=5, respectively), and tumor formation and lymph node metastasis were examined on days 8, 15 and 22, using an *in vivo* imaging system (Figure 3B). In three independent experiments, we found the metastasis rate is higher in SAS_Luc2_ASC mice than in SAS_Luc2 mice (*p*=0.033) (Figure 3C). Anatomy result demonstrated that the tumor cells metastasized to the cervical-region lymph nodes of mice injected with SAS_Luc2_ASC cells (Figure 3D). IHC staining confirmed that ASC was overexpressed in tongue and lymph node sections from mice carrying SAS_Luc2_ASC tumors but not in those obtained from SAS_Luc2 control mice (Figure 3E). In terms of mortality, the SAS_Luc2 and SAS_Luc2_ASC groups respectively experienced the deaths of one and four mice by day 15, and five and two additional mice by day 22 (these mice were examined for tumor metastasis at death and included in our analysis). These results suggest that ASC-overexpressing tumor cells may have enhanced potential for metastasis.

## DISCUSSION

Here, we show that the NLRP3 inflammasome-related proteins, ASC, IL-1β, CASP1, and NLRP3, are all highly expressed in OSCC tumor cells compared to adjacent normal cells. Of them, ASC was significantly correlated with various clinicopathological characteristics (Table 1), including node stage, overall stage [31], extracapsular spread, and perineural invasion, and was an independent predictor of OS, DSS, and DFS for OSCC patients (Table 2). *In vitro* and *in vivo* studies further demonstrated that ASC appears to promote tumor metastasis. These results are highly consistent with clinical observations of OSCC examined in this study. Above all, overexpression of ASC in OSCC is highly positively correlated with OSCC tumor progression. To our knowledge, this is the first report to show that ASC has oncogenic activity in OSCC.

In this context, ASC gene expression is reportedly down-regulated through DNA methylation of its promoter, mainly within the region spanning nts −146 to +246. In contrast to the prior reports, we herein found that ASC was overexpressed in OSCC tumor tissues. We examined the DNA methylation status of the ASC gene promoter within this region by bisulfide sequencing in two paired OSCC/normal adjacent biopsies, which showed high ASC expression by IHC. As presented in Supplementary Figure S2A, we confirmed that the 37 CpG sites within the nts −146 to +246 region of the ASC gene promoter were unmethylated. In addition, Western blot analysis confirmed that ASC was more highly expressed in the tumor tissues compared to the paired adjacent normal tissues (Supplementary Figure S2B). IHC staining of the 111 paired OSCC samples revealed that only two failed to show expression of ASC. Moreover, ASC overexpression in tumor cells was also detected in Epstein-Barr virus-associated NPC [22]. Further studies will be needed to determine the mechanism(s) responsible for regulating ASC expression in OSCC.

In this study, high level of ASC expression in OSCC specimens predicted poorer prognosis of patients, which is different from a previously report by Shimane *et al.* [31]. The difference can be resulted from the following reasons. First, the scoring methods used in our study and their study were different. In our study, the scores for each IHC specimens were multiplied by the percentage of cells that showed positive staining, and the scores reflected protein expression (Materials and methods). The resulting scores were used to classify the specimens/patients into two groups: ‘high-level’ protein expression and ‘low-level’ protein expression. For ASC, scores ≤120 was defined as low level, scores >120 defined as high level. For the other study, the scoring method of IHC results to classify specimens was based on the intensity of anti-ASC antibody alone, and specimens were classified into level 1, 2 or 3. Second, another difference may be due to the age distribution of patients; the average age of OSCC was 49.5 years old and the ratio of male:female was 7:1, reflecting the age and gender distribution of OSCC in Taiwan, whereas the average age was 65 years old and male: female ratios was 1.3:1 in that study.

Inflammasome activation has been reported in many kinds of cancer, including liver cancer [32], gastric cancer [33], NPC [22] and melanoma [29]. However, no study to date has investigated the potential involvement of inflammasome activity in OSCC. Our microarray data and RT-qPCR results revealed that other pattern recognition receptors, such as AIM2 and RIG-I, also showed elevated expression in OSCC tumor tissues (data not shown). The microbiome including bacteria species in oral cavity are altered in tumor and non-tumorous tissues as revealed by the next generation sequencing studies [34, 35]. Thus, microorganisms or their products may activate cells to generate hydrogen peroxide, which in turn activates NLRP3 inflammasome, and to induce DNA damage to activate AIM2 inflammsome in epithelial cells [36]. Using OSCC cell lines, NLRP3 and other inflammasomes can be induced, suggesting of activated inflammsomes in OSCC (data not shown) by factors in microenvironement. These results indicated that inflammasome activity may be induced by pathogens in OSCC. Inflammasome activation has been reported in other pathogen-related cancers, such as in HCV-associated liver cancer [37], *Helicobacter*-associated gastric cancer [33] and EBV-associated NPC [22]. In oral cancer, HPV and few oral bacteria are thought to be related to OSCC progression [34, 38, 39]. However, we do not yet know if these pathogens induce inflammasome activation, and if so, whether this inflammasome activation is associated with OSCC. Smoking and betel quid chewing have been strongly associated with OSCC progression in Taiwan [40]. However, we observed that neither nicotine nor arecoline triggered inflammasome activation, as assessed by measuring the level of secreted IL-1β (data not shown). Considering the diversity and complexity of the microbes found in oral saliva [34], other factors or combinations of multiple factors may be involved in OSCC.

In *in vivo* oral metastasis experiment, we recorded the IVIS data of all experimental mice, but we were unable to collect tongue, lymph node, lung and liver specimens from mice those died during the experiment. To determine lymph node metastasis of tumor, we performed the IHC analysis using anti-ASC antibody on tongue, lymph node, lung and liver specimens from the IVIS-positive mice. For subject No.14 (Figure 3A at d22), IVIS was negative but IHC showed positive result (Supplementary Figure S3). In addition, we did not observe lung or liver metastasis in these mice (data not shown). There was no significant difference in survival rate between SAS_Luc2 mice and SAS_ Luc2_ASC mice (data not shown). Thus, tumor-related *in vivo* results here support that ASC promotes metastasis of OSCC cells.

In this study, only ASC, but not the other inflammasome-associated molecules NRLP3, CASP1 and IL-1β was strongly associated with the clinicopathological characteristics (e.g. tumor stage and node stage) and was an independent predictor of OS, DSS, and DFS of OSCC patients. In *in vitro* and *in vivo* studies, ASC induced cell migration and invasion as well as induced metastasis in mice. We have tested the effect of ASC on E-cadherin, a key epithelial-mesenchymal transition (EMT) gene (unpublished results). We found that E-cad gene expression were decreased 65% in SAS_Luc2_ASC cells compared to SAS_Luc2 cells using qRT-PCR analysis. This result indicates that ASC may mediate cell migration and invasion through regulating EMT genes such as E-cadherin. We are currently planning additional studies to explore the molecular mechanism(s) underlying the ASC-mediated promotion of metastasis in OSCC.

In summary, we herein use clinical samples, cell lines and *in vivo* studies to show that ASC can act as a potential biomarker of metastasis in OSCC. Recently, small molecule inhibitors have been developed against ASC [41]. Given our present findings, we propose that ASC may be a potential oncotarget for OSCC therapy.

## MATERIALS AND METHODS

### Cell culture

The SAS cell line (Bioresource Collection and Research Center, Taiwan) was maintained in DMEM supplemented with 10% fetal bovine serum (FBS). OC3 cells derived from buccal epidermal carcinoma [42] were maintained in keratinocyte serum-free medium (Invitrogen, Carlsbad, CA, USA) and DMEM/10% FBS (2:1 ratio). OECM1 cells derived from gingival epidermal carcinoma [43] were maintained in RPMI supplemented with 10% FBS. All three cell lines were obtained from Professor Jau-Song Yu in Graduate Institute of Biomedical Sciences, Chang Gung University, Tao-Yuan, Taiwan.

### Patient information

Clinical samples were obtained from a consecutive cohort of 111 OSCC patients for IHC analysis and 20 OSCC patients for RT-quantitative PCR exam diagnosed at the Chang Gung Memorial Hospital (Tao-Yuan, Taiwan) from August 2002 to June 2007. OSCC patients presenting with unresectable disease, synchronous cancers, distant metastasis, or any previous history of malignancy were excluded. All patients provided informed consent prior to their participation, and this study was approved by the Institutional Review Board. According to the institution's guidelines, each patient underwent a standard preoperative work-up that included a detailed medical history, a complete physical examination, computed tomography or magnetic resonance imaging scans of the head and neck, chest radiographs, a bone scan, and an abdominal ultrasound. Primary tumors were intraoperatively excised with adequate margins under frozen-section control. Classic radical or modified neck dissection (levels I–V) was performed in patients with clinically positive lymph node disease. Supraomohyoid neck dissection (levels I–III) was performed in clinically node-negative patients [44]. When necessary, surgical defects were immediately reconstructed by plastic surgeons using free or local flap techniques. The pathological and nodal stages of all tumors were established as described in the AJCC Cancer Staging Manual (2010). Post-operative radiotherapy was performed within 6 weeks following surgery on patients with pathologic T4 tumors and positive lymph nodes. Patients with pathologic evidence of multiple neck lymph node metastasis and/or extracapsular spread received concurrent adjuvant chemoradiotherapy (a cisplatin-based regimen plus a total radiation dose of 66 Gy given as 1.8-2 Gy per day for 5 days per week). After discharge, all patients had regular follow-up visits every 2 months for the first year, every 3 months for the second year, and every 6 months thereafter [45, 46].

### Bisulfite sequencing

Genomic DNA (2μg) was modified by sodium bisulfite using an EZ DNA methylation kit (Zymo Research, USA). The promoter region of *asc* from nts −146 to +246 (containing 37 CpG dinucleotide sites) was amplified with specific primers (5′-TTA GGT AGA AGT TGA TTA GTT TGT-3′ and 5′-CCA AAA ACC TAA ATA AAA AAA A-3′). The PCR products were cloned into the TA vector (YEASTERN BIOTECH. CO., LTD) and sequenced. The sequencing results were analyzed using Vector NTI 9.0 software (Invitrogen, USA).

### Immunohistochemical staining of OSCC specimens

IHC analyses were performed using a Bondmax automated immunostainer (Bond, Vision BioSystems) according to the manufacturer's instructions. An anti-ASC antibody (1:100; MBL International Corporation) was used to detect ASC in clinical tissue sections and mouse tongues and lymph nodes. Standard staining and IHC scoring methods were used, as previously described [22]. Briefly, IHC intensity was divided into four graded as 0, 1, 2, or 3 to indicate undetectable, weak, moderate, and strong staining, respectively. The scores for each IHC specimens were multiplied by the percentage of cells that showed positive staining, and the scores reflected protein expression. The resulting scores were used to classify the specimens/patients into two groups: ‘high-level’ protein expression and ‘low-level’ protein expression. For ASC, scores ≤120 was defined as low level, scores >120 was defined as high level; for CASP1, score ≤100 was low level, and >100 was high level; for IL-1β, score ≤40 was low level, and >40 was high level; for NLRP3, score ≤160 was low, and >160 was high.

### Lentivirus construction and establishment of stable clones

Three functional plasmids were used, as follows: (1) pLKO.AS2.neo/pLKO.AS2.ASC (1 μg), representing the control and ASC-overexpressing vectors, (2) helper plasmid pCMV-ΔR8 (0.9 μg), which expressed the viral *gag* and *pol* genes, and (3) envelope plasmid pMD.G (0.9 μg). Lentiviruses were constructed according to the provided protocol and co-transfected into HEK293 cells for packaging (the National RNAi Core Facility of Academia Sinica). To establish a stably transfected clone, SAS cells were transfected with ready-to-use lentiviruses containing a firefly luciferase 2 (Luc2) reporter gene [47] and infected with pLKO.AS2.neo or pLKO.AS2.ASC lentivirus in 6 μg/ml of Polybrene (Sigma-Aldrich) and selected with 400 μg/ml Geneticin (Life Technologies) and 2 μg/ml puromycin (Life Technologies). The selected clones SAS_Luc2 and SAS_Luc2_ASC were subjected to cell proliferation assays and used for our animal studies.

### RNA extraction and RT-quantitative (q)PCR

Total RNA was isolated from the oral cancer cell lines using the TRIZOL reagent (Invitrogen) and cDNA was generated with SuperScript III (Invitrogen) according to the manufacturer's protocol. Real-time qPCR was performed using Light-cycler (Roche). The expression of GAPDH was monitored as an internal control. The examined genes and primers used in this study were described as below: *il-1b*, 5′-TCT CCG ACC ACC ACT AC-3′ and 5′-AGC CTC GTT ATC CCA T-3′; *asc*, 5′-ATC CAG GCC CCT CCT CAG T-3′ and 5′-GTT TGT GAC CCT CGC GAT AAG-3′; *casp1*, 5′-GAA TGT CAA GCT TTG CTC CCT AGA-3′ and 5′- AAG ACG TGT GCG GCT TGA CT-3′; *nlrp3*, 5′-TCT GTG TGT GGG ACT GAA GCA-3′ and 5′-TAC TGA TGC AAG ATC CTG ACA ACA-3′.

### Cell proliferation assay

SAS (2×10^5^) or OECM1 (2×10^5^) cells were seeded to six-well culture plates and transiently transfected with plasmids pLKO.neo.AS2 or pLKO.neo.AS2.ASC for ASC-overexpression studies; with scrambled or ASC-targeting siRNAs (Dharmacon, GE) for ASC knockdown studies. Cell numbers were counted daily for 4 days. Each test was performed in duplicate at least three independent times.

### Trans-well migration assay

Trans-well migration assays were performed in a 24-well Trans-well chamber (Corning, USA) fitted with a polycarbonate membrane (8-μm pore size), as previously described [48]. Briefly, SAS (1×10^5^), OECM1 (1×10^5^) or OC3 (2×10^5^) cells were washed two or three times with serum-free medium, re-suspended in 100 μl of serum-free medium, and seeded to the upper chamber. The lower chamber was loaded with medium containing 10% FBS, and the plates were cultured for 16 hours under 5% CO_2_ at 37°C. The migrated cells attached on the chamber membrane were fixed and stained with 0.25% crystal violet, 10% formaldehyde and 80% methanol for 15 minutes, and then washed five times with ddH_2_O. The migrated cells were enumerated by averaging the counts obtained from 10 random microscopic fields under 100× magnification. The test was performed three independent times.

### Cell invasion

Invasion assays were performed using a Chemicon cell invasion assay kit (ECM550; Millipore, MA, USA). Briefly, the ECM layer in the insert (8-μm pore size) was rehydrated in serum-free medium (300μl) for 2 hours at room temperature. The medium was then replaced with 300μl serum-free medium containing 1×10^6^ cells. Medium containing 10% FBS was added to the lower chamber, and the cells were incubated at 37°C under 5% CO_2_ for 24 hours. The non-invaded cells were gently removed with a cotton swab, and the invaded cells were fixed and stained for 15 minutes with 0.25% crystal violet, 10% formaldehyde and 80% methanol. The non-adherent cells were removed by five washes with ddH_2_O, and the remaining cells were counted and averaged from 10 random microscopic fields (100× magnification). The test was performed three independent times.

### Animal study

Nude mice (8 to 12 weeks old) were obtained from National Laboratory Animal Center. For animal study, we have performed three independent experiments; 5 mice were inoculated with SAS_Luc2 control cells and 5 mice were inoculated with SAS_Luc2_ASC cells in each experiment. We then analyzed the number of mice with lymph node metastasis in control vs. ASC-expressing group from each experiment, independently. The statistical significance was determined by student *t* test between these two groups. Briefly, SAS_Luc2 and SAS_Luc2_ASC cell lines (2.5×10^5^ in 40 μl serum-free DMEM) were inoculated into the tip of the mouse tongue [49–51]. Tumor progression was monitored once a week, using an IVIS Spectrum (Xenogen IVIS 100; Caliper) [47]. Twenty-two days later, mice were sacrificed, and tongue and cervical region lymph nodes were subjected to IHC examination using anti-ASC antibody.

### Statistical analysis

All statistical data are expressed as means ± SD. The Wilcoxon signed ranks test was used to compare the relative signal intensities (IHC staining scores) of paired tumor and pericancerous normal epithelium samples. Cell proliferation, migration, invasion and lymph node metastasis (in our animal study) were compared using the *Student's t-test*. The associations of various clinicopathological parameters with the IHC scores for ASC, IL-1β, and CASP1 were evaluated using the Wilcoxon test. All statistical analyses were performed using the SAS software (version 9.1; SAS Institute Inc., Cary, NC). All patients received follow-up evaluations at our outpatient clinic until October 2011 or death. The survival time and various time intervals were calculated from the date of operation. Survival analyses were plotted using the Kaplan-Meier method, and differences were evaluated with the log-rank test. Univariate and multivariate regression analyses were performed under the Cox proportional hazard model, and were employed to define specific risk factors for survival status. All *p* values were two-sided, and statistical significance was accepted at *p* < 0.05.

## SUPPLEMENTARY MATERIALS FIGURES AND TABLES



## References

[1] Parkin DM, Bray F, Ferlay J, Pisani P (2005). Global cancer statistics, 2002. CA Cancer J Clin.

[2] Reid BC, Winn DM, Morse DE, Pendrys DG (2000). Head and neck in situ carcinoma: incidence, trends, and survival. Oral oncology.

[3] Funk GF, Karnell LH, Robinson RA, Zhen WK, Trask DK, Hoffman HT (2002). Presentation, treatment, and outcome of oral cavity cancer: a National Cancer Data Base report. Head & neck.

[4] Diaz EM, Holsinger FC, Zuniga ER, Roberts DB, Sorensen DM (2003). Squamous cell carcinoma of the buccal mucosa: one institution's experience with 119 previously untreated patients. Head & neck.

[5] Muir C, Weiland L (1995). Upper aerodigestive tract cancers. Cancer.

[6] Wong YK, Tsai WC, Lin JC, Poon CK, Chao SY, Hsiao YL, Chan MY, Cheng CS, Wang CC, Wang CP, Liu SA (2006). Socio-demographic factors in the prognosis of oral cancer patients. Oral oncology.

[7] Scully C, Bagan JV (2007). Recent advances in Oral Oncology. Oral oncology.

[8] Zhen W, Karnell LH, Hoffman HT, Funk GF, Buatti JM, Menck HR (2004). The National Cancer Data Base report on squamous cell carcinoma of the base of tongue. Head & neck.

[9] Kodani I, Shomori K, Osaki M, Kuratate I, Ryoke K, Ito H (2001). Expression of minichromosome maintenance 2 (MCM2), Ki-67, and cell-cycle-related molecules, and apoptosis in the normal-dysplasia-carcinoma sequence of the oral mucosa. Pathobiology.

[10] Lippman SM, Sudbo J, Hong WK (2005). Oral cancer prevention and the evolution of molecular-targeted drug development. Journal of clinical oncology.

[11] Silverman S, Gorsky M, Lozada F (1984). Oral leukoplakia and malignant transformation. A follow-up study of 257 patients. Cancer.

[12] Sudbo J (2004). Novel management of oral cancer: a paradigm of predictive oncology. Clinical medicine & research.

[13] Pauwels NS, Bracke KR, Dupont LL, Van Pottelberge GR, Provoost S, Vanden Berghe T, Vandenabeele P, Lambrecht BN, Joos GF, Brusselle GG (2011). Role of IL-1alpha and the Nlrp3/caspase-1/IL-1beta axis in cigarette smoke-induced pulmonary inflammation and COPD. Eur Respir J.

[14] Chang LY, Wan HC, Lai YL, Kuo YF, Liu TY, Chen YT, Hung SL (2009). Areca nut extracts increased expression of inflammatory cytokines, tumor necrosis factor-alpha, interleukin-1beta, interleukin-6 and interleukin-8, in peripheral blood mononuclear cells. Journal of periodontal research.

[15] Lippai D, Bala S, Petrasek J, Csak T, Levin I, Kurt-Jones EA, Szabo G (2013). Alcohol-induced IL-1beta in the brain is mediated by NLRP3/ASC inflammasome activation that amplifies neuroinflammation. Journal of leukocyte biology.

[16] Masumoto J, Taniguchi Si, Ayukawa K, Sarvotham H, Kishino T, Niikawa N, Hidaka E, Katsuyama T, Higuchi T, Sagara J (1999). ASC, a novel 22-kDa protein, aggregates during apoptosis of human promyelocytic leukemia HL-60 cells. Journal of Biological Chemistry.

[17] Srinivasula SM, Poyet J-L, Razmara M, Datta P, Zhang Z, Alnemri ES (2002). The PYRIN-CARD protein ASC is an activating adaptor for caspase-1. Journal of Biological Chemistry.

[18] Martinon F, Burns K, Tschopp J (2002). The inflammasome: a molecular platform triggering activation of inflammatory caspases and processing of proIL-beta. Mol Cell.

[19] Okamoto M, Liu W, Luo Y, Tanaka A, Cai X, Norris DA, Dinarello CA, Fujita M (2010). Constitutively active inflammasome in human melanoma cells mediating autoinflammation via caspase-1 processing and secretion of interleukin-1beta. The Journal of biological chemistry.

[20] Hu B, Elinav E, Huber S, Booth CJ, Strowig T, Jin C, Eisenbarth SC, Flavell RA (2010). Inflammation-induced tumorigenesis in the colon is regulated by caspase-1 and NLRC4. Proc Natl Acad Sci U S A.

[21] Patsos G, Germann A, Gebert J, Dihlmann S (2010). Restoration of absent in melanoma 2 (AIM2) induces G2/M cell cycle arrest and promotes invasion of colorectal cancer cells. International journal of cancer.

[22] Chen LC, Wang LJ, Tsang NM, Ojcius DM, Chen CC, Ouyang CN, Hsueh C, Liang Y, Chang KP, Chang YS (2012). Tumour inflammasome-derived IL-1beta recruits neutrophils and improves local recurrence-free survival in EBV-induced nasopharyngeal carcinoma. EMBO Mol Med.

[23] Guan X, Sagara J, Yokoyama T, Koganehira Y, Oguchi M, Saida T, Taniguchi S (2003). ASC/TMS1, a caspase-1 activating adaptor, is downregulated by aberrant methylation in human melanoma. International journal of cancer.

[24] Conway KE, McConnell BB, Bowring CE, Donald CD, Warren ST, Vertino PM (2000). TMS1, a novel proapoptotic caspase recruitment domain protein, is a target of methylation-induced gene silencing in human breast cancers. Cancer research.

[25] Akahira J, Sugihashi Y, Ito K, Niikura H, Okamura K, Yaegashi N (2004). Promoter methylation status and expression of TMS1 gene in human epithelial ovarian cancer. Cancer Sci.

[26] Das PM, Ramachandran K, Vanwert J, Ferdinand L, Gopisetty G, Reis IM, Singal R (2006). Methylation mediated silencing of TMS1/ASC gene in prostate cancer. Molecular cancer.

[27] Riojas MA, Guo M, Glockner SC, Machida EO, Baylin SB, Ahuja N (2007). Methylation-induced silencing of ASC/TMS1, a pro-apoptotic gene, is a late-stage event in colorectal cancer. Cancer Biol Ther.

[28] Zhang C, Li H, Zhou G, Zhang Q, Zhang T, Li J, Zhang J, Hou J, Liew CT, Yin D (2007). Transcriptional silencing of the TMS1/ASC tumour suppressor gene by an epigenetic mechanism in hepatocellular carcinoma cells. J Pathol.

[29] Liu W, Luo Y, Dunn JH, Norris DA, Dinarello CA, Fujita M (2013). Dual role of apoptosis-associated speck-like protein containing a CARD (ASC) in tumorigenesis of human melanoma. The Journal of investigative dermatology.

[30] Wu CS, Chang KP, Chen LC, Chen CC, Liang Y, Hseuh C, Chang YS (2012). Heterogeneous ribonucleoprotein K and thymidine phosphorylase are independent prognostic and therapeutic markers for oral squamous cell carcinoma. Oral oncology.

[31] Shimane T, Kobayashi H, Takeoka M, Kitazawa M, Matsumura T, Hida S, Xiao T, Koike T, Taniguchi S, Kurita H (2013). Clinical significance of apoptosis-associated speck-like protein containing a caspase recruitment domain in oral squamous cell carcinoma. Oral surgery, oral medicine, oral pathology and oral radiology.

[32] Burdette D, Haskett A, Presser L, McRae S, Iqbal J, Waris G (2012). Hepatitis C virus activates interleukin-1beta via caspase-1-inflammasome complex. The Journal of general virology.

[33] Semper RP, Mejias-Luque R, Gross C, Anderl F, Muller A, Vieth M, Busch DH, Prazeres da Costa C, Ruland J, Gross O, Gerhard M (2014). Helicobacter pylori-induced IL-1beta secretion in innate immune cells is regulated by the NLRP3 inflammasome and requires the cag pathogenicity island. Journal of immunology.

[34] Pushalkar S, Mane SP, Ji X, Li Y, Evans C, Crasta OR, Morse D, Meagher R, Singh A, Saxena D (2011). Microbial diversity in saliva of oral squamous cell carcinoma. FEMS immunology and medical microbiology.

[35] Wang L, Ganly I (2014). The oral microbiome and oral cancer. Clinics in laboratory medicine.

[36] Vogelmann R, Amieva MR (2007). The role of bacterial pathogens in cancer. Current opinion in microbiology.

[37] Negash AA, Ramos HJ, Crochet N, Lau DT, Doehle B, Papic N, Delker DA, Jo J, Bertoletti A, Hagedorn CH, Gale M (2013). IL-1beta production through the NLRP3 inflammasome by hepatic macrophages links hepatitis C virus infection with liver inflammation and disease. PLoS pathogens.

[38] Mager DL, Haffajee AD, Devlin PM, Norris CM, Posner MR, Goodson JM (2005). The salivary microbiota as a diagnostic indicator of oral cancer: a descriptive, non-randomized study of cancer-free and oral squamous cell carcinoma subjects. Journal of translational medicine.

[39] Gupta S, Gupta S (2015). Role of human papillomavirus in oral squamous cell carcinoma and oral potentially malignant disorders: A review of the literature. Indian journal of dentistry.

[40] Chen PC, Kuo C, Pan CC, Chou MY (2002). Risk of oral cancer associated with human papillomavirus infection, betel quid chewing, and cigarette smoking in Taiwan–an integrated molecular and epidemiological study of 58 cases. Journal of oral pathology & medicine.

[41] Gross CJ, Gross O (2015). The Nlrp3 inflammasome admits defeat. Trends Immunol.

[42] Lin SC, Liu CJ, Chiu CP, Chang SM, Lu SY, Chen YJ (2004). Establishment of OC3 oral carcinoma cell line and identification of NF-kappa B activation responses to areca nut extract. Journal of oral pathology & medicine.

[43] Meng CL, Chao CF, Tu CL, Chang I.C (1984). Establishment, and characterization of a human oral epidermoid carcinoma cell line. Clin. Dent. J.

[44] Liao CT, Chang JT, Wang HM, Ng SH, Hsueh C, Lee LY, Lin CH, Chen IH, Kang CJ, Huang SF, Tsai MF, Yen TC (2006). Surgical outcome of T4a and resected T4b oral cavity cancer. Cancer.

[45] Fang KH, Kao HK, Cheng MH, Chang YL, Tsang NM, Huang YC, Lee LY, Yu JS, Hao SP, Chang KP (2009). Histological differentiation of primary oral squamous cell carcinomas in an area of betel quid chewing prevalence. Otolaryngology–head and neck surgery.

[46] Chang KP, Yu JS, Chien KY, Lee CW, Liang Y, Liao CT, Yen TC, Lee LY, Huang LL, Liu SC, Chang YS, Chi LM (2011). Identification of PRDX4 and P4HA2 as metastasis-associated proteins in oral cavity squamous cell carcinoma by comparative tissue proteomics of microdissected specimens using iTRAQ technology. Journal of proteome research.

[47] Liu SC, Tsang NM, Chiang WC, Chang KP, Hsueh C, Liang Y, Juang JL, Chow KP, Chang YS (2013). Leukemia inhibitory factor promotes nasopharyngeal carcinoma progression and radioresistance. The Journal of clinical investigation.

[48] Wu CS, Lu YJ, Li HP, Hsueh C, Lu CY, Leu YW, Liu HP, Lin KH, Hui-Ming Huang T, Chang YS (2010). Glutamate receptor, ionotropic, kainate 2 silencing by DNA hypermethylation possesses tumor suppressor function in gastric cancer. International journal of cancer.

[49] Shintani S, Mihara M, Nakahara Y, Aida T, Tachikawa T, Hamakawa H (2002). Lymph node metastasis of oral cancer visualized in live tissue by green fluorescent protein expression. Oral oncology.

[50] Myers JN, Holsinger FC, Jasser SA, Bekele BN, Fidler IJ (2002). An orthotopic nude mouse model of oral tongue squamous cell carcinoma. Clinical cancer research.

[51] Hung PS, Liu CJ, Chou CS, Kao SY, Yang CC, Chang KW, Chiu TH, Lin SC (2013). miR-146a enhances the oncogenicity of oral carcinoma by concomitant targeting of the IRAK1, TRAF6 and NUMB genes. PloS one.

