# Supplementary material for: ASC contributes to metastasis of oral cavity squamous cell carcinoma
Source: Oncotarget. 2016 Jun 29;7(31):50074–85. doi: 10.18632/oncotarget.10317 (PMC5226569; doi:10.18632/oncotarget.10317)
Supplement: Supplementary file 1 [file oncotarget-07-50074-s001.pdf]

ASC contributes to metastasis of oral cavity squamous cell carcinoma

SUPPLEMENTARY MATERIALS AND METHODS

Microarray analysis

Tumor samples of various sites in oral cavity including 48 OSCC (44 men and 4 women) and 14 adjacent normal clinical samples (diagnosed at the Chang Gung Memorial Hospital, Tao-Yuan, Taiwan) were subjected to microarray analysis (Affymetrix Gene Chip HG-U133A). Demographic characteristics of Patients including age, gender, tumor sites, and tumor stages are listed in Supplementary Table S2.

SUPPLEMENTARY FIGURES AND TABLES

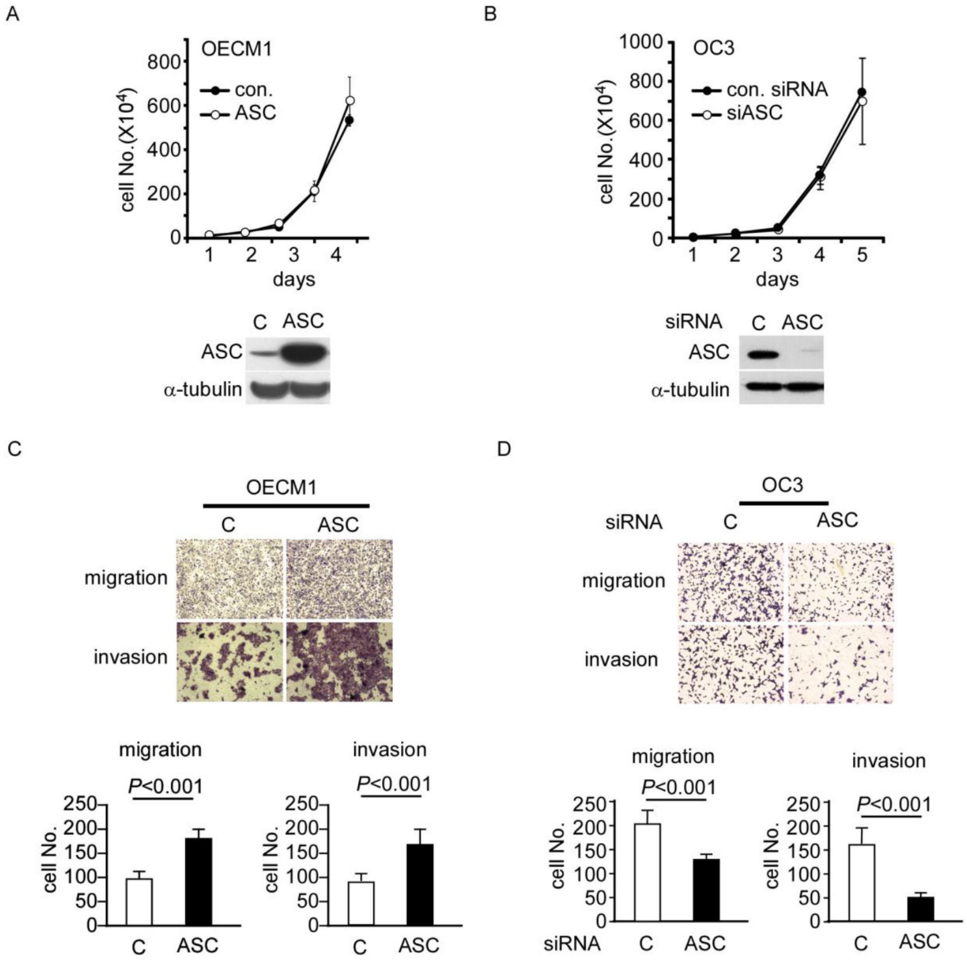

Supplementary Figure S1: Tumor-related parameters in ASC-overexpressing OSCC cell lines. A. OECM1 cells transiently expressing ASC or the vector (control) were monitored for cell proliferation over 4 days. **B.** OC3 cells with and without *asc*-specific siRNA-mediated knockdown were monitored for cell proliferation. Western blot analysis confirms the up- and down-regulations of ASC expression in the tested OECM1 and OC3 cells. **C.** OECM1 cells transiently expressing ASC or the vector (control) were assessed for migration (upper) and invasion (lower). Quantifications of migration and invasion are shown at the right. **D.** OC3 cells with and without siRNA-mediated knockdown of ASC were assessed for migration (upper) and invasion (lower). Quantifications of migration and invasion are shown at the right.

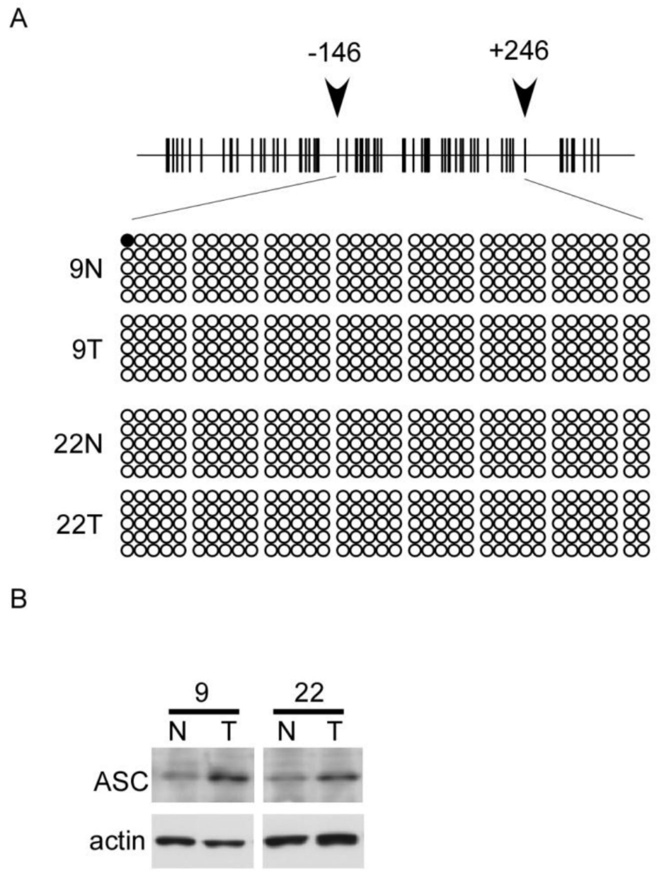

Supplementary Figure S2: CpG island methylation in the promoter region of ASC. A. Genomic DNA from paired tumor (T)/ normal (N) samples of two OSCC patients was analyzed for the methylation of 37 CpG sites in the *asc* gene promoter region (nts -146 to +246). Each circle represents one CpG site. **B.** Western blot analysis confirms the ASC were overexpression in tumor tissues (T) than in adjacent normal tissues (N).

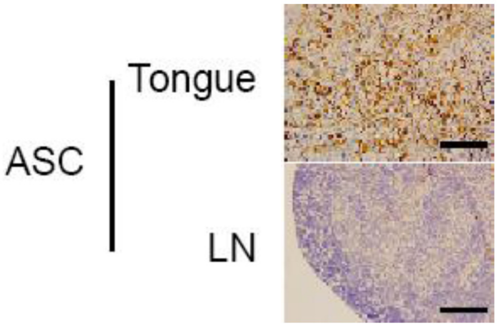

Supplementary Figure S3: IHC results of subject No.14 mice in the animal study. ASC were detected in tongue but weekly stained in lymph node region. Magnification, X200; bar, 100 μm.

Supplementary Table S1: Characteristics of 111 patients with OSCC

Characteristics	Subjects number
Gender	
Male	97
Female	14
Age (years)	
mean±SD	50.0±11.8
Tumor Location	
Buccal	44
Tongue	45
Gingiva	9
Mouth floor	5
Lip	5
Hard palate	3
Overall TNM stage	
Stage I	13
Stage II	29
Stage III	14
Stage IV	55
Total	111

Supplementary Table S2: Characteristics of 48 OSCC patients for microarray analysis

Characteristics	Subjects number
Gender	
Male	44
Female	4
Age (years)	
mean±SD	51.3±9.7
Tumor Location	
Buccal	27
Tongue	14
Gum	5
Uvula	2
Overall TNM stage	
Stage I	1
Stage II	15
Stage III	5
Stage IV B	5
Stage IV A	22
Total	48
